# Primary pure spindle cell carcinoma (sarcomatoid carcinoma) of the ovary: A case report with immunohistochemical study

**DOI:** 10.1186/s13000-016-0521-3

**Published:** 2016-08-05

**Authors:** Giovanna Giordano, Roberto Berretta, Enrico Silini

**Affiliations:** 1 Department of Biomedical, Biotechnological and Translational Sciences, Pathological Anatomy and Histology Unit, Faculty of Medicine, University of Parma, Parma, Italy; 2 Department of Obstetric and Gynecologic Sciences and Neonatology, Parma University, Parma, Italy

**Keywords:** Malignant mixed Müllerian tumors, Sarcomatoid carcinoma, Carcinosarcoma, Monophasic sarcomatoid tumor, Biphasic tumor

## Abstract

**Background:**

In the ovary, sarcomatoid carcinoma has been reported only as mural nodules in epithelial malignant or borderline serous or mucinous cystic neoplasms, and in teratomas.

In this paper we report a rare case of a solid sarcomatoid carcinoma of the ovary, without accompanying component of giant cells, pleomorphic cells, or glandular and other epithelial structures.

**Case presentation:**

This case report refers to a sarcomatoid carcinoma of the ovary in in a 57 year-old woman with abdominal pain.

Macroscopically, the neoplasm was a 15x10x5 cm ovarian mass that featured gray white solid fleshy areas, interspersed with areas of necrosis, hemorrhage and cystic spaces filled with thick fluid. The epithelial differentiation of the tumor was demonstrated by strong and diffuse reactivity to CAM5.2 and focal immunoreactivity to EMA. A diagnosis of malignant mesenchymal tumor was excluded due to negativity for desmin, smooth muscle actin, caldesmon, CD34, CD10, and myoglobin. Neural, neuroendocrine neoplasm, melanoma and Perivascular Epithelioid Cell Tumor (PEComa) were excluded because of negativity for S100, chromogranin, synaptophysin and HMB45.

**Conclusion:**

Primary ovarian spindle cell carcinoma is a rare neoplasm, which must be considered in the differential diagnosis of solid ovarian mass with spindle cell appearance. This case adds to our knowledge of the biological behavior of these rare neoplasms. The distinction from true sarcomas and carcinosarcomas is important because of the more favorable prognosis of the spindle cell carcinomas. However their diagnosis necessitates a careful tissue sampling and immunohistochemical staining.

## Background

Sarcomatoid carcinoma is form of cancer, with controversial histogenesis, which shares histological, cytological, or molecular properties of both epithelial and mesenchymal differentiation.

Sarcomatoid carcinoma is referred to a pure spindle cell carcinoma which is distinguished from true sarcoma by its positive staining to cytokeratins. Multiple sections and immunohistochemical stains or ultrastructural study are required to support the diagnosis [1, 2].

“The carcinosarcoma” is a neoplasm with carcinomatous and sarcomatous elements [3, 4].

Sarcomatoid carcinomas, known also as spindle cell (sarcomatoid) carcinomas, are rare malignancies and have been reported in many organs such as the breast [3, 5] urinary bladder [1, 2, 6], kidney [7] and lung [4, 8]. Ovarian anaplastic spindle cell carcinomas include three different subtypes, rhabdoid, spindled (sarcomatoid) and pleomorphic in epithelial malignant [9, 10], or serous and mucinous borderline [11, 12] cystic neoplasms, and in teratomas [13].

In this paper, we report a rare case of a solid sarcomatoid carcinoma of the ovary of spindle cell type.

## Case presentation

A 53-year-old woman presented to our institution complaining of abdominal pain. Her personal history was unremarkable. Physical examination revealed a palpable mass lesion in the pelvic region. The results of laboratory investigations revealed increased CA125 levels (176.56 U/ml) (normal value: < 35 U/ml). Pelvic magnetic resonance imaging (MRI) scans suggested a malignant lesion located in the left ovary, showing the presence a large, complex mass with cystic solid components and necrotic areas. Hysterectomy with bilateral salpingo-oophorectomy, pelvic lymph node dissection and omentectomy were performed to establish stage of development of this neoplasm.

### Pathologic findings

**Fig. 1:**
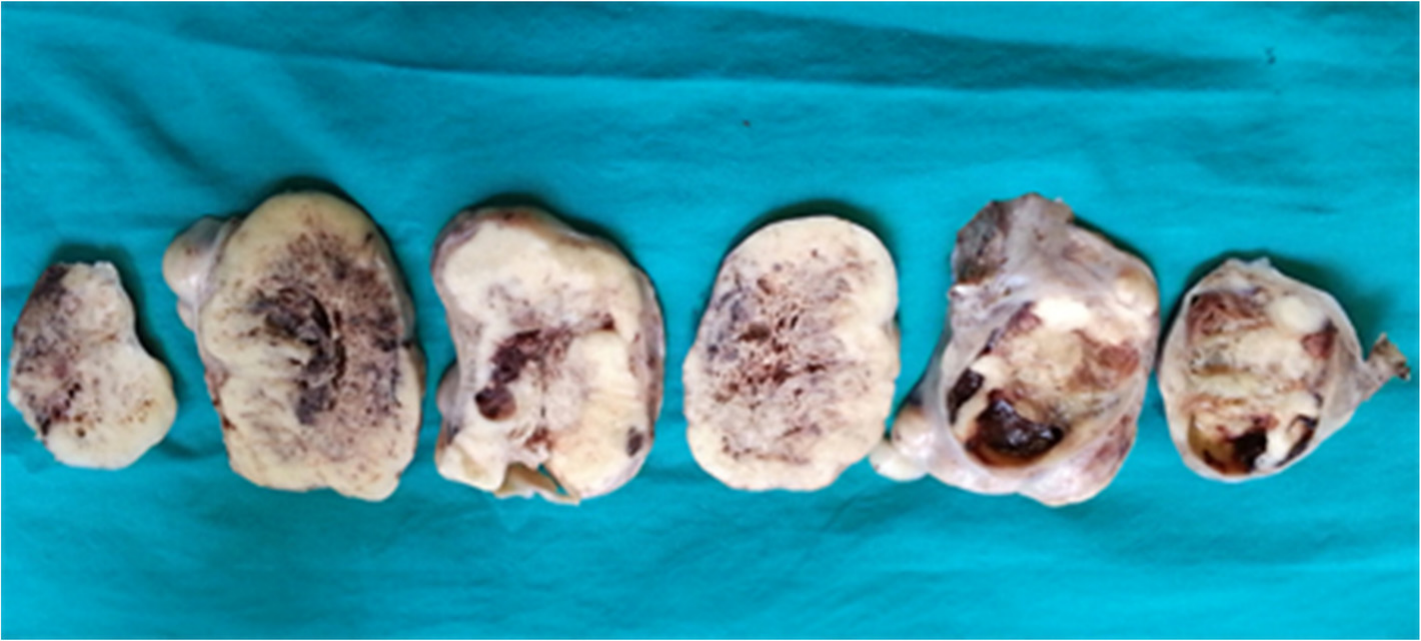
Left salpingo-oophorectomy specimen showing a 15x10x5 cm ovarian mass with gray white solid fleshy areas, interspersed with areas of necrosis, hemorrhage, and cystic spaces

On macroscopic examination, a left salpingo-oophorectomy specimen revealed an intact 15x10x5 cm ovarian mass. A cut section showed gray white solid fleshy areas, interspersed with areas of necrosis, hemorrhage, and cystic spaces filled with thick fluid (Fig. 1). Cystic spaces were entirely submitted for histological evaluation.

For accurate microscopic examination of solid areas, we used a sampling, suggested by other investigators for diagnosis of mucinous borderline and malignant tumors of the ovary [14]. In line with this protocol, sampling of our tumor could be considered as optimal, because was examined one section per 1 cm of the maximum diameter [14].

**Fig. 2:**
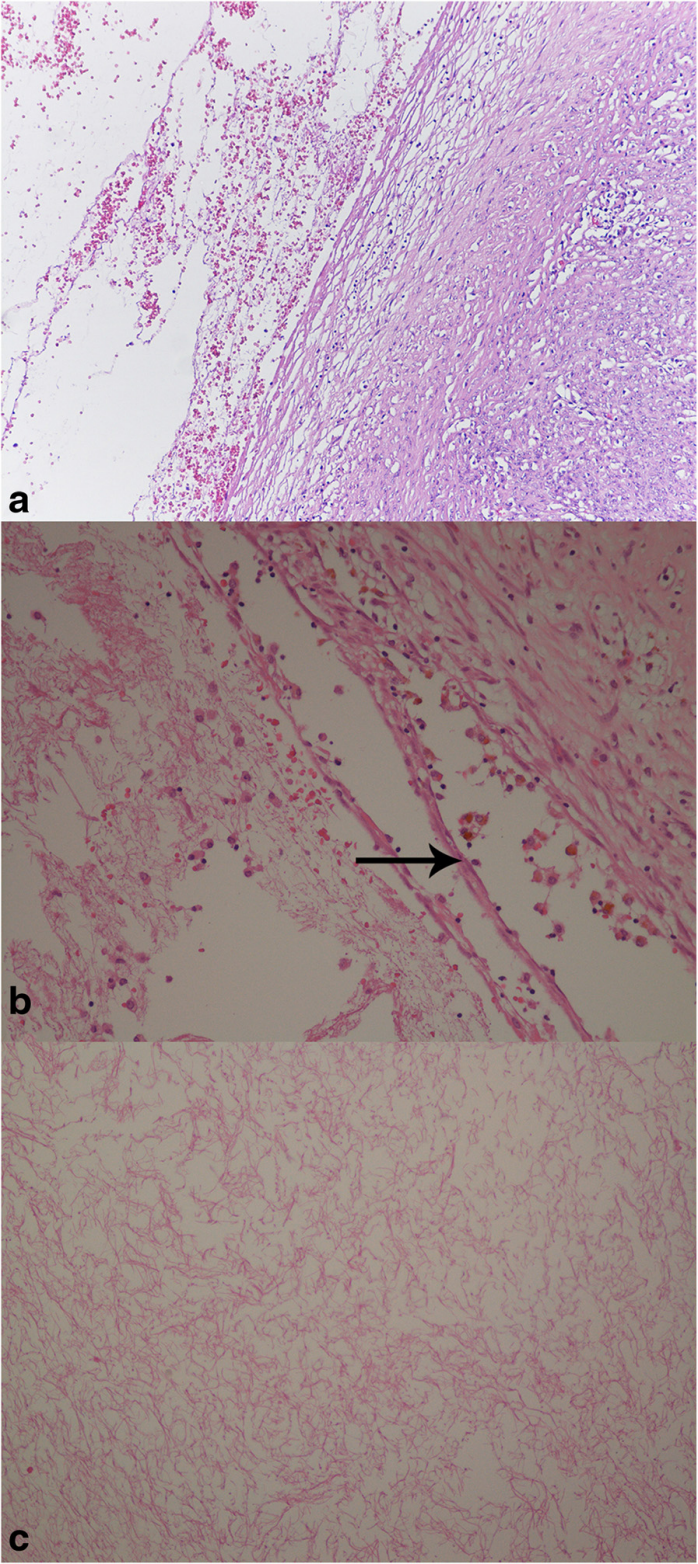
R1: Microscopic examination revealed that the cystic spaces showed no epithelial lining (**a**: Hematoxylin-eosin x 100). Note the presence of macrophages filled with hemosiderin in the wall of these spaces (**b**: Hematoxylin-eosin, *Arrow* x 200) and fibrin with pink network aspect in the cavity of cystic space (**c**: Hematoxylin-eosin, x 200)

Microscopic examination revealed that the cystic spaces, which were entirely submitted for histological evaluation, showed no epithelial lining (Fig. 2a) and that they corresponded to edematous and hemorrhagic areas due to the torsion of mass. In fact, the wall of these spaces contained macrophages filled with hemosiderin (Fig. 2b). The thick fluid observed macroscopically in these spaces was not mucous material, but it corresponded to fibrin, with pink network aspect (Fig. 2c).

**Fig. 3:**
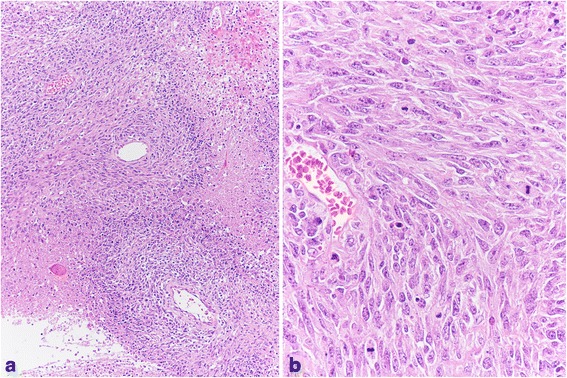
The solid component of the neoplasm contained large foci of coagulative tumor cell necrosis and spindle cells, with moderate cytoplasm, arranged predominantly in fascicles or a storiform pattern, mimicking mesenchymal malignancy (**a**: Hematoxylin-eosin x 100). In other smaller areas, the neoplastic elements showed abundant eosinophilic cytoplasm. The nuclei were atypical and vesicular, with evident nucleoli. Mitoses were frequent and sometimes atypical (**b**: Hematoxylin-eosin x 400)

The solid component of the neoplasm contained large foci of coagulative tumor cell necrosis and predominantly spindle cells, with moderate cytoplasm, arranged in fascicles or a storiform pattern, mimicking mesenchymal malignancy (Fig. 3a).

In other smaller areas, the spindle neoplastic elements showed more abundant cytoplasm with epithelial-like appearance. The nuclei were atypical and vesicular, with evident nucleoli. Mitoses were frequent and sometimes atypical (Fig. 3b).

In numerous sections examined, no benign or malignant epithelial structures, nor histological elements, such as giant cells or pleomorphic cells were observed.

Immunohistochemical analysis was performed to establish true nature of tumor differentiating epithelial from mesenchymal, neuroendocrine neoplasms, melanoma and uterine Perivascular Epithelioid Cell Tumor (PEComa), sex cord tumors and (Gastrointestinal Stromal tumor) GIST.

**Fig. 4:**
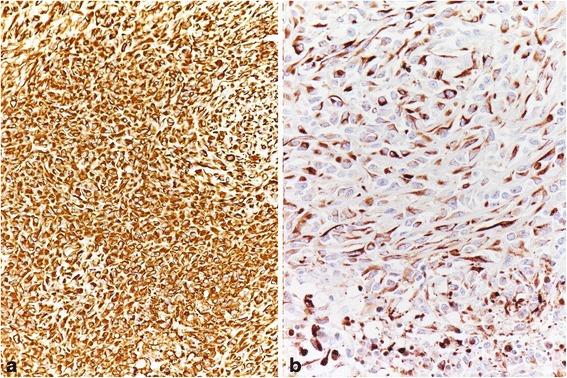
On immunohistochemistry, the tumor cells showed diffuse strong positive immunoreactivity for Vimentin (**a**: Vimentin x 200) and strong positive reaction for CAM 5.2 (**b**: CAM 5.2 x 400)

**Fig. 5:**
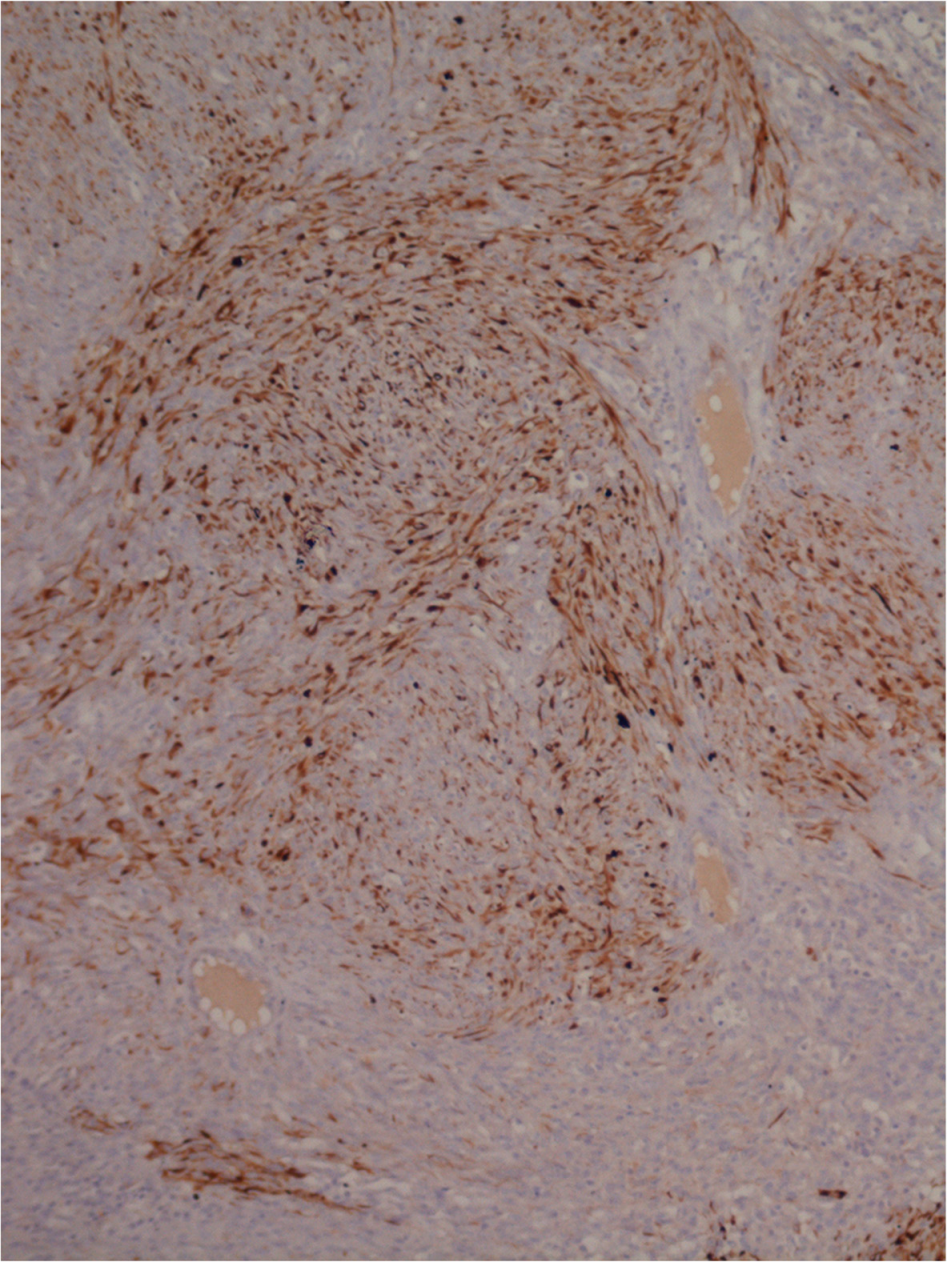
On immunohistochemistry, the tumor cells showed focal positivity to EMA (x 100)

On immunohistochemistry, the tumor cells showed strong and diffuse reactivity to Vimentin (Fig. 4a), CAM5.2 (Fig. 4b) and focal positivity to cocktail of keratins and EMA (Fig. 5). Immunoreactivity was negative when staining for other antibodies such as Ca 125, desmin, smooth muscle actin, S100, caldesmon, CD34, calretinin, alpha-inhibin, CD10, myoglobin, S100 protein, HMB45, chromogranin, synaptophysin, and c-kit.

Although a carcinomatous component was not recognized at microscopic examination, the epithelial nature of the tumor was established because of strong and diffuse reactivity to CAM 5.2 and focal positivity to EMA and cocktail of keratins.

Specimen of pelvic lymph node dissection did not reveal presence of metastases.

Abdominal ultrasound, chest X-ray, total computed tomography, or bone scan were unremarkable.

Therefore, the final diagnosis was primary ovarian sarcomatoid carcinoma of pT1a N0 M0 stage of development at the diagnosis.

The patient received six cycles of chemotherapy with carboplatin-Taxol.

After one year from diagnosis, surgery and chemotherapy, the patient is free of disease.

## Conclusions

Ovarian carcinosarcomas, also known as malignant mixed mesodermal tumors or malignant mixed Müllerian tumors, are exceedingly rare, and comprise only 1-3 % of ovarian malignancies [14, 15].

Histologically, ovarian carcinosarcomas are considered biphasic tumors with both carcinomatous (epithelial) and sarcomatous (stromal) elements. They can be sub-classified as “heterologous” or “homologous” based on the presence or absence of a stromal component containing mesenchymal tissues not normally found at the primary tumour site.

Spindle cell carcinoma is defined as a spindle cell neoplasm, that simulates a sarcoma and demonstrates epithelial differentiation in both immunohistochemistry and electron microscopy [1, 2, 4–8].

Anaplastic carcinoma of spindle cell type is an exceedingly rare ovarian neoplasm, with controversial histogenesis, with less than ten cases reported in the English medical literature. Three different types have been described, rhabdoid, spindled (sarcomatoid) and pleomorphic [10].

To the best of our knowledge, in the ovary, sarcomatoid carcinoma has only been reported as mural nodules in epithelial malignant [9, 10], or borderline [11, 12] cystic neoplasms, and in teratomas [13].

In our case, sarcomatoid carcinoma of the ovary was characterized only by a spindle cell component without epithelial structures.

In this case, the epithelial differentiation of the tumor was demonstrated by strong and diffuse reactivity to CAM 5.2 and focal positivity to EMA. A diagnosis of malignant mesenchymal tumor was excluded due to negativity for desmin, smooth muscle actin, caldesmon, CD34, CD10, and myoglobin. Neural, neuroendocrine neoplasm and melanoma and uterine Perivascular Epithelioid Cell Tumor (PEComa) were excluded because of negativity for S100, chromogranin, synaptophysin and HMB45.

Other neoplams such as sex cord tumors and (Gastrointestinal Stromal tumor) GIST were excluded due to negativity to negativity for alpha-Inhibin, and C-kit.

In many organs such as the kidney [16, 17], breast [3], and urinary bladder [1] sarcomatoid carcinomas are neoplasms, with poor prognosis. As a consequence of the relative rarity and diagnostic heterogeneity of these tumors, it has proven difficult to properly predict their behaviour and to determine optimal management.

Although it was first thought to carry an invariably unfavorable prognosis, recent data indicate that this does not apply to Ia tumors [10].

Our findings are consistent with the above mentioned data. In this case, at diagnosis, the neoplasm was located within the ovary (Stage: pT1a), without invasion of surrounding tissue and lymph nodes metastases and was treated with combined chemotherapy treatment with carboplatin and taxol. After one year from diagnosis, the patient is free of disease and this might suggest that its aggressiveness is lower than anaplastic spindle cell carcinoma, present in other organs.

The sarcomas are characterized by presence of malignant mesenchymal elements and these include stromal cell sarcomas, fibrosarcomas, leiomyosarcomas, neurofibrosarcomas, rhabdomyosarcomas, chondrosarcomas, angiosarcomas, and liposarcomas [18]. Carcinosarcoma is a mixed Müllerian (MMT), and is characterized by the presence of both carcinomatous and sarcomatous component. Ovarian MMTs can be classified as homologous or heterologous. In the homologous subtype can be observed tissue normally present in the ovary. On the contrary, in the heterologous subtype the neoplasm shows tissues that is not normally present in the ovary [19].

Extensive sampling is mandatory for the exclusion of MMTs, which carry a more ominous prognosis [19].

Our case refers to a spindle cell carcinoma of the ovary which appears as a solid mass. For diagnosis in this case cystic spaces were entirely submitted for histological evaluation and solid areas were examined using protocol used by other Author for diagnosis of mucinous borderline and malignant tumors of the ovary [14].

Furthermore, in our opinion, this case, is in line with other cases of sarcomatoid carcinomas observed in different organs.

This is the first case report of sarcomatoid (spindle cell carcinoma) of the ovary, which appears as a solid mass. Moreover, this case demonstrates that findings of malignant spindle cell proliferation does not imply that this entity is a sarcoma or malignant Mixed Müllerian Tumor (MMT). Additionally, careful tissue sampling and immunohistochemical analysis to distinguish between these different entities is mandatory.

## Abbreviations

GIST, gastro-intestinal stromal tumor; MMT, mixed Müllerian tumor; MRI, magnetic resonance imaging; PEComa, Perivascular Epithelioid Cell Tumor

## References

[1] Lopez-Beltran A, Pacelli A, Rothenberg HJ, Wollan PC, Zincke H, Blute ML (1998). Carcinosarcoma and sarcomatoid carcinoma of the bladder: clinicopathological study of 41 cases. J Urol.

[2] Cheng L, Zhang S, Alexander R, Maclennan GT, Hodges KB, Harrison BT (2011). Sarcomatoid carcinoma of the urinary bladder: the final common pathway of urothelial carcinoma dedifferentiation. Am J Surg Pathol.

[3] Foschini MP, Dina RE, Eusebi V (1993). Sarcomatoid neoplasms of the breast: proposed definitions for biphasic and monophasic sarcomatoid mammary carcinomas. Semin Diagn Pathol.

[4] Nappi O, Glasner SD, Swanson PE, Wick MR (1994). Biphasic and monophasic sarcomatoid carcinomas of the lung. A reappraisal of ‘carcinosarcomas’ and “spindle-cell carcinomas”. Am J Clin Pathol.

[5] Carter MR, Hornick JL, Lester S, Fletcher CD (2006). Spindle cell (sarcomatoid) carcinoma of the breast: a clinicopathologic and immunohistochemical analysis of 29 cases. Am J Surg Pathol.

[6] Torenbeck R, Blomjous CEM, Bruin PC, Newling DWW, Meijer CLJM (1994). Sarcomatoid cancer of the urinary bladder. Clinicopathological analysis of 18 cases with immunohistochemical and electron microscopic findings. Am J Surg Pathol.

[7] Molina AM, Tickoo SK, Ishill N, Trinos MJ, Schwartz LH, Patil S (2011). Sarcomatoid-variant renal cell carcinoma: treatment outcome and survival in advanced disease. Am J Clin Oncol.

[8] Terada T (2010). Spindle cell carcinoma of the lung: Frequency, clinical features, and immunohistochemical studies of three cases. Respi Med CME.

[9] Czernobilsky B, Dgani R (1983). Roth L Ovarian mucinous cistoadenocarcinoma with mural nodule of carcinomatous derivation. Cancer.

[10] Hillesheim PB, Farghaly H (2010). Anaplastic spindle cell carcinoma, arising in a background of an ovarian mucinous cystic tumor: a case report with clinical follow up, review of the literature. Int J Clin Exp Pathol.

[11] Dutton PM, Beattie G, Al-Nafussi A (2008). Sarcomatoid carcinoma arising within a serous borderline ovarian tumour: a case report and practical approach to differential diagnosis. Histopathology.

[12] Ghosh P, Saha K, Bhowmik S (2014). Sarcoma-like mural nodule in a borderline mucinous tumor of the ovary: A rare entity. J Midlife Health.

[13] Czernobilsky B, Rotenstreich L, Lancet M (1972). Ovarian dermoid with squamous carcinoma-pseudosarcoma. Arch.

[14] Guerrieri C, Högberg T, Wingren S, Fristedt S, Simonsen E, Boeryd B (1994). Mucinous borderline and malignant tumors of the ovary. A clinicopathologic and DNA ploidy study of 92 cases. Cancer.

[15] Mano MS, Rosa DD, Azambuja E, Ismael G, Braga S, D’Hondt V (2007). Current management of ovarian carcinosarcoma. Int J Gynecol Cancer.

[16] Peralta-Venturina M, Moch H, Amin M, Tamboli P, Hailemariam S, Mihatsch M (2001). Sarcomatoid differentiation in renal cell carcinoma: A study of 101 cases. Am J Surg Pathol.

[17] Cheville JC, Lohse CM, Zincke H, Weaver AL, Leibovich BC, Frank I (2004). Sarcomatoid renal cell carcinoma: An examination of underlying histologic subtype and an analysis of associations with patient outcome. Am J Surg Pathol.

[18] Sahin A, Benda JA (1988). An immunohistochemical study of primary ovarian sarcoma. An evaluation of nine tumors. Int J Gynecol Pathol.

[19] Menon S, Deodhar K, Rekhi B, Dhake R, Gupta S, Ghosh J (2013). Clinico-pathological spectrum of primary ovarian malignant mixed mullerian tumors (OMMMT) from a tertiary cancer institute: A series of 27 cases. Indian J Pathol Microbiol.

